# The Cost of the Cure: Antibiotic Exposure as a Risk Factor for Irritable Bowel Syndrome

**DOI:** 10.3390/antibiotics15080772

**Published:** 2026-08-11

**Authors:** Abdulrahman Ismaiel, Mhd Bashir Almonajjed, Ahmed Abdelghafar, Mahdi Wardeh, Simona Grad, Teodora Surdea-Blaga, Stefan-Lucian Popa, Mohamed Ismaiel, Mohamed Abosheisha, Andreas-Friedrich Krauss, Paul Grama, Simona Bataga, Dan L. Dumitrascu

**Affiliations:** 1 2nd Department of Internal Medicine, “Iuliu Hatieganu” University of Medicine and Pharmacy, 400006 Cluj-Napoca, Romania; 2 Faculty of Medicine, “Iuliu Hatieganu” University of Medicine and Pharmacy, 400006 Cluj-Napoca, Romania; m.basheralmounajed@gmail.com (M.B.A.); ahmedabdelghafar2002@gmail.com (A.A.); mahdi.wardeh2003@gmail.com (M.W.); 3 General Surgery Department, University Hospital Limerick, V94 F858 Limerick, Ireland; 4 General Surgery Department, Wirral University Teaching Hospital NHS Trust, Wirral CH49 5PE, UK; 5 Faculty of Medicine, “Victor Babes” University of Medicine and Pharmacy, 300041 Timisoara, Romania; 6 M3 Department, Discipline of Internal Medicine 1, George Emil Palade University of Medicine, Pharmacy, Science, and Technology of Târgu Mureș, 540139 Targu Mures, Romania; 7 Department of Gastroenterology, Târgu Mures County Emergency Clinical Hospital, 540136 Targu Mures, Romania; 8 Romanian Academy of Scientists, 050044 Bucharest, Romania

**Keywords:** Irritable Bowel Syndrome (IBS), risk factors, dysbiosis, antibiotic exposure, Disorders of Gut–Brain Interaction (DGBI)

## Abstract

The intricate interplay between the gut microbiome and the enteric nervous system remains a paramount focus in understanding the multifactorial pathogenesis of disorders of gut–brain interaction (DGBI), most notably irritable bowel syndrome (IBS). While the clinical entity of post-infectious IBS is well-established, the independent, long-term pathophysiological impact of iatrogenic antibiotic exposure is garnering critical attention within neurogastroenterology. This narrative review provides a comprehensive synthesis of current epidemiological and mechanistic evidence positioning antibiotic-induced microbial depletion as a potential predisposing factor for incident IBS. By evaluating recent literature, we highlight epidemiological trends demonstrating a consistent, dose-dependent relationship between cumulative antibiotic courses, particularly broad-spectrum agents, and an elevated risk of developing IBS, independent of prior acute enteric infections. Furthermore, we explore the mechanistic underpinnings of this association, focusing on how systemic antibiotics induce persistent, detrimental alterations in commensal diversity. This resulting dysbiosis initiates a proposed cascade of downstream consequences, including compromised epithelial barrier integrity, persistent low-grade mucosal inflammation, and altered bile acid metabolism. These localized disruptions serve as established triggers for visceral hypersensitivity and dysregulated gastrointestinal motility communicated via the gut–brain axis. Ultimately, this review underscores that antibiotic exposure may act as a significant, modifiable risk factor for IBS pathogenesis. Recognizing this substantial iatrogenic risk reinforces an urgent clinical imperative for stringent antimicrobial stewardship and emphasizes the necessity for future research directed toward prophylactic, microbiome-sparing strategies to mitigate the escalating global burden of DGBIs.

## 1. Introduction

Historically, Irritable Bowel Syndrome (IBS) was classified as a functional gastrointestinal disorder, a designation that often obscured the underlying pathophysiological disruptions. While the Rome IV criteria initiated a paradigm shift a decade ago, the recently published Rome V criteria have firmly cemented the reclassification of these conditions as Disorders of Gut–Brain Interaction (DGBIs) [1,2,3]. The Rome V framework intentionally retires the outdated “functional” terminology to reduce stigma and accurately reflect the disease’s complex physiological and neurogastroenterological basis [3]. This reclassification acknowledges IBS as a complex, multifactorial disease involving bidirectional dysregulation among the central nervous system (CNS), the enteric nervous system (ENS), the mucosal immune system, and the luminal microbiome [1,3]. DGBIs impose a substantial epidemiological burden, affecting over 40% of the global population across 26 countries in internet surveys, reducing quality of life (QoL), and straining healthcare systems due to increasing demand for diagnostics and specialist care [4].

The classical pathophysiological understanding of acquired IBS has long been dominated by post-infectious IBS (PI-IBS). Seminal clinical investigations span several decades. They demonstrated that around 11% of patients suffering an acute bout of bacterial, viral, or protozoal gastroenteritis subsequently develop chronic IBS symptoms [5]. Pathogens such as *Campylobacter jejuni*, *Salmonella*, and *Shigella* cause extensive mucosal damage. This precipitates an acute inflammatory cascade characterized by intraepithelial T lymphocytes infiltration, elevated interleukin-1β expression, and the proliferation of serotonin-secreting enterochromaffin (EC) cells [6,7,8]. This pathogen-driven acute injury results in a lasting molecular signature that disrupts ENS sensory afferents [6,7]. This produces prolonged visceral hypersensitivity, causing altered bowel habits to persist long after the infectious agent is neutralized [7,9,10].

Although the PI-IBS model is well-characterized and widely accepted, recent neurogastroenterological research has introduced a new paradigm: iatrogenic, antibiotic-associated IBS. Historically, systemic antibiotics were considered to cause only temporary disturbances in the host ecosystem [11]. However, high-resolution metagenomic sequencing has demonstrated that these agents inflict significant, long-term collateral damage to the commensal gut microbiota [11]. This antibiotic-induced microbial depletion is referred to as “microbiome scarring”. It is increasingly recognized as a potential predisposing factor for the development of IBS [12,13,14].

Rather than representing a distinct clinical diagnosis, antibiotic-associated IBS risk serves as a proposed pathophysiological framework. While PI-IBS arises from pathogen-induced mucosal destruction, this iatrogenic model is hypothesized to result from the pharmacological eradication of essential commensal phyla, though clinically separating these overlapping triggers remains challenging [13,14]. The resulting loss of taxonomic diversity deprives the colonic epithelium of microbe-derived short-chain fatty acids (SCFAs) and disrupts biochemical networks involved in bile acid (BA) recycling, tight junction integrity, and mucosal immune tolerance [15,16]. A critical evaluation of this phenomenon is required because systemic antimicrobial exposure is increasingly recognized as a potential pathogenic contributor. Thus, the primary aims of this review are to summarize the existing epidemiological evidence establishing broad-spectrum antibiotic exposure as an independent risk factor for the incidence of IBS, describe the mechanistic cascade of “microbiome scarring” and its downstream neuroimmune consequences on the gut–brain axis, and emphasize the urgent clinical significance of such findings, underscoring the need for strict antimicrobial stewardship and implementation of prophylactic, microbiome-sparing therapeutic approaches. To inform this narrative review, a comprehensive literature search was conducted using the PubMed/MEDLINE and Scopus electronic databases without formal restrictions on publication date. The search strategy utilized combinations of Medical Subject Headings (MeSH) and free-text keywords, including but not limited to: *“irritable bowel syndrome”*, *“IBS”*, *“antibiotics”*, *“microbiome”*, *“dysbiosis”*, *“bile acids”*, *“visceral hypersensitivity”*, *“intestinal permeability”*, and *“gut–brain axis”*. To evaluate the validity of this hypothesis, it is first necessary to examine the population-level data. Section 2 explores the epidemiological evidence linking systemic antibiotic exposure to the subsequent onset of IBS.

## 2. Epidemiological Evidence: Antibiotic Exposure as an Independent Risk Factor

The hypothesis that antibiotic exposure precipitates functional bowel symptoms has been rigorously examined over the past decade through large-scale, population-level epidemiological studies. Using comprehensive national health registries and well-controlled longitudinal cohorts, researchers have delineated the temporal relationship between systemic antimicrobial use and subsequent development of IBS, thereby minimizing confounding variables and strengthening evidence for a robust temporal association.

Krogsgaard et al. conducted a foundational study utilizing a prospectively followed Danish cohort, which yielded some of the earliest population-based evidence linking antibiotic usage to subsequent development of functional bowel disorders [12]. The researchers surveyed asymptomatic individuals representative of the Danish general population and monitored their transition from gastrointestinal health to meeting Rome III criteria for IBS over a three-year period. Within the cohort, 22.4% reported antibiotic consumption in the preceding year. Notably, the incidence of new-onset IBS was significantly higher among this exposed group; individuals initially asymptomatic who had been exposed to antibiotics demonstrated a relative risk (RR) of 1.9 and a 95% confidence interval (CI) of 1.1 to 3.1 for developing IBS compared to unexposed controls [12]. This longitudinal evidence demonstrated that antibiotic exposure frequently precedes symptom onset in previously healthy individuals, thus fulfilling the epidemiological criterion of temporality.

The extent of this risk was subsequently validated, expanded upon, and meticulously quantified by Staller et al., who performed a large-scale nationwide case–control study utilizing the highly detailed Swedish Patient Register and Prescribed Drug Register [13]. By analyzing data from 29,111 adult patients newly diagnosed with IBS and 135,172 matched controls, they observed that 74.9% of IBS patients had received systemic antibiotic prescriptions in the year preceding their diagnosis, compared to just 57.8% of controls. After applying rigorous multivariable adjustments, prior antibiotic use was associated with a markedly increased odds of developing IBS, with an odds ratio (OR) of 2.21 (95% CI: 2.14 to 2.28) [13]. Additionally, an exclusionary buffer period of one year prior to diagnosis was implemented to mitigate reverse causation, ensuring that antibiotics were not prescribed in response to early, misdiagnosed IBS symptoms [13].

A significant finding from the Swedish registry data was the identification of a consistent, linear dose-dependent relationship between cumulative antibiotic exposure and the risk of developing IBS. Patients who received 1 to 2 antibiotic dispensations exhibited an OR of 1.67 (95% CI 1.61 to 1.73), whereas those receiving 3 or more dispensations showed an OR of 3.36 (95% CI 3.24 to 3.49) (*p* for trend < 0.001) [13]. This pronounced dose–response gradient supports a potential biological association, suggesting that repeated ecological disruptions progressively diminish the resilience of the gut microbiome. With each additional antibiotic course, the ecosystem’s resilience is progressively challenged, ultimately contributing to the pathogenesis of chronic DGBI in susceptible individuals [11,13,17]. Importantly, the increased risk persisted across different antibiotic classes, implying that the overall extent of broad-spectrum microbial depletion, rather than specific chemical toxicity, is the principal pathogenic factor [13].

Despite these findings, a prevalent epidemiological critique argues that the initial infection prompting antibiotic administration, rather than the medication itself, may serve as the actual physiological trigger, thus confounding antibiotic-associated IBS risk with classical PI-IBS [11,13,17]. Furthermore, establishing a direct link between antibiotic use and subsequent IBS is inherently challenging. When evaluating patients who develop symptoms after antimicrobial treatment, it is difficult to isolate the effects of the antibiotics from confounding variables, including the underlying infection itself, previously existing but undiagnosed gastrointestinal symptoms, inherent healthcare-seeking behavior, concomitant medications, and underlying psychosocial factors [14]. To address this issue, a pivotal nested case–control study conducted in Olmsted County, Minnesota, effectively disentangled this confounder [14]. Researchers examined the onset of functional gastrointestinal disorders (FGID) specifically following non-enteric infections (such as upper respiratory, urinary tract, or dermatological infections) [14]. Their analysis indicated that 83% of newly diagnosed FGID cases had recently experienced a non-enteric infection treated with systemic antibiotics. Logistic regression analysis demonstrated that antibiotic therapy for non-gastrointestinal infections was independently associated with an increased OR of 1.90 (95% CI: 1.21 to 2.98, *p* = 0.005) for developing a subsequent DGBI [14]. Since these infections did not directly involve or inflame the gastrointestinal mucosa, the data effectively isolate the iatrogenic impact of circulating antimicrobial agents on the enteric microbiome, thereby clearly separating it from pathogen-driven damage characteristic of acute gastroenteritis.

A comprehensive systematic review and meta-analysis conducted by Colecchia et al. analyzed data from 31 studies involving a total of 422,350 patients. The study found an overall pooled incidence of IBS of 26% among antibiotic users, compared to 20% in non-users, demonstrating an incidence rate ratio (IRR) of 1.30 (95% CI: 1.07 to 1.58) [18]. Notably, the meta-analysis revealed a synergistic interaction when antibiotics were administered concurrently with an acute enteric infection [18]. Specifically, antibiotic use aimed at treating gastrointestinal infections was associated with a significantly higher risk of developing IBS (IRR 1.71; 95% CI: 1.16 to 2.51) [18]. These results support a “double-hit” hypothesis: the enteric pathogen causes acute structural damage to the mucosa, while the concurrent use of broad-spectrum antibiotics destroys the commensal flora essential for epithelial repair and immune resolution, thereby markedly increasing the risk of progression to chronic PI-IBS [15,18].

The socioeconomic and clinical impact of antibiotic-associated IBS is considerable. Longitudinal studies utilizing data from the United Kingdom Clinical Practice Research Datalink (CPRD) reveal that the overprescription of antibiotics in primary care settings remains widespread [19]. Patients diagnosed with IBS following systemic antibiotic exposure tend to utilize healthcare services chronically over prolonged periods. Following diagnosis, these patients average nearly £1000 in excess annual healthcare expenditures [19,20]. Furthermore, their QoL follows a distinct trajectory: while patients typically experience an improvement for the first three months after visiting a gastroenterologist, their QoL ultimately declines after one year [21]. A comprehensive summary of these foundational studies, including their designs and primary risk metrics, is detailed in Table 1. While epidemiological studies establish a strong associative risk, the severity of this risk is not uniform across all medications. Section 3 explores how the specific pharmacological spectrum of an antibiotic dictates the extent of ecological disruption.

## 3. The Spectrum of Damage: Broad-Spectrum Antibiotics and the Threshold of Ecological Disruption

The specific pharmacological imprint of the antimicrobial drug used determines the epidemiological relationship between antimicrobial treatment and the development of new-onset IBS. Recent population-level registry data highlight a marked difference in the iatrogenic etiology of DGBIs, which is substantially influenced by the antibiotic spectrum used. A nationwide case–control study reveals that while all assessed antibiotic classes increase the odds of developing IBS, specific agents carry higher risks [13]. Tetracyclines present the greatest association with an adjusted Odds Ratio (OR) of 2.18 (95% CI: 2.11 to 2.24) [13]. This is followed closely by quinolones (OR 2.16; 95% CI: 2.08 to 2.25) and macrolides (OR 2.06; 95% CI: 1.98 to 2.13) [13]. In contrast, narrower-spectrum agents such as penicillins demonstrate a comparatively lower risk profile (OR 1.72; 95% CI: 1.67 to 1.77) [13]. Furthermore, this risk exhibits a strict dose-dependent trajectory regardless of the antibiotic class [13]. Compared to unexposed individuals, patients with 1 to 2 historical antibiotic dispensations face an increased risk (OR 1.67; 95% CI: 1.61 to 1.73), while those receiving 3 or more prescriptions exhibit a substantially higher risk (OR 3.36; 95% CI: 3.24 to 3.49) (*p* for trend < 0.001) [13]. Broad-spectrum antibiotics, particularly fluoroquinolones, macrolides, and broad-spectrum beta-lactams, cause indiscriminate ecological disruption, resulting in substantial and long-term taxonomic depletion in the lower gastrointestinal tract [17]. In contrast to narrow-spectrum treatments, which impose highly focused selection pressure while essentially conserving commensal networks, broad-spectrum regimens eradicate keystone obligate anaerobes indiscriminately.

Among these bacterial deaths are crucial SCFA producers, especially *Faecalibacterium prausnitzii* and *Clostridium* clusters IV and XIVa [22]. Butyrate and other SCFAs serve as the metabolic lifeblood of colonocytes and are essential for sustaining the expression of key tight junction proteins, particularly Zonula Occludens-1 (ZO-1) and Occludin, meaning that their rapid removal damages the structural integrity of the mucosal barrier [23,24,25]. This disturbance increases paracellular permeability, allowing luminal antigens and endotoxins to enter the submucosa, resulting in low-grade neuroimmune inflammation and visceral hypersensitivity [24,26,27].

Crucially, this antimicrobial collateral damage is not temporary; rather, it follows a strict, dose-dependent trajectory that results in chronic microbiome scarring [11,13]. A healthy gut microbiota has innate immunological and metabolic resilience, allowing it to withstand small alterations [17]. However, repeated treatments of broad-spectrum antibiotics steadily destroy this resilience, reducing functional redundancy and microbial cooperation [11,17]. With each subsequent antibiotic exposure, the bar for ecological recovery raises [17]. This dysbiotic configuration, characterized by decreased SCFA synthesis, loss of colonization resistance, and the proliferation of opportunistic pathobionts (e.g., Enterobacteriaceae), alters host physiology in a self-perpetuating cycle of mucosal inflammation, defining the pathophysiological core of DGBIs [28]. Narrow-spectrum agents, on the contrary, preserve the commensal community’s critical mass, minimizing collateral scarring and permitting the ecosystem to return to its original, homeostatic stable condition before reaching the tipping point [15].

The impact of antimicrobial agents on the risk of IBS needs to be considered since not all antibiotics have the same impact on the host microbiome. The ecological result is basically defined by the specific activity spectrum, systemic absorption, pharmacokinetics and duration of treatment [13,17]. An example is the use of rifaximin as a first-line therapeutic agent for certain IBS phenotypes, in a paradoxical use of a minimally absorbed oral antibiotic. It is strongly recommended currently in clinical guidelines for the treatment of IBS-D [29]. Rifaximin has a distinctive eubiotic effect. Although it exhibits a broad antimicrobial spectrum in vitro, its lack of systemic absorption and its solubility in a specific microenvironment allow it to selectively modulate the gut microbiome, decreasing mucosal inflammation and altering bacterial fermentation, without the significant, long-term dysbiosis of highly absorbed systemic antibiotics [30,31]. This highlights that while some systemic antibiotic exposures predispose to increased IBS risk, targeted, non-absorbable agents may successfully reduce IBS symptoms. The indiscriminate eradication of commensal taxa by broad-spectrum agents can push the microbiome beyond its capacity to recover. Section 4 examines the specific ecological mechanisms of this “microbiome scarring” and how it fundamentally alters the gut environment.

## 4. Mechanistic Underpinnings: The Cascade of “Microbiome Scarring”

Understanding the impact of systemic, transient antibiotic exposure on the development of persistent, chronic functional bowel disorders necessitates an examination of microbial ecology principles, including the concept of the “alternative stable state”. The adult human gut microbiome represents a highly complex ecosystem that generally maintains a state of dynamic equilibrium, supporting symbiotic homeostasis with the host’s enteric nervous and immune systems. Intentional disturbances, such as the administration of broad-spectrum antibiotics, can induce a substantial decline in biodiversity, thereby fundamentally modifying the spatial and metabolic architecture of the gut lumen [11,32].

The foundational research by Dethlefsen and Relman systematically delineates the temporal dynamics of this ecological disruption [11,32]. Their methodology involved administering repeated courses of the broad-spectrum fluoroquinolone ciprofloxacin to healthy human volunteers, followed by the analysis of millions of 16S rRNA hypervariable region sequences over a period of ten months. Their findings indicated a rapid and significant reduction in microbial taxonomic diversity within three to four days of antibiotic administration [11]. Although there was substantial interindividual variability, the pattern of microbial decline was universally observed. Importantly, while the microbiome exhibited a recovery phase post-antibiotic cessation, the reversion to the original baseline was persistently incomplete. Instead of reinstating the previous ecological equilibrium, the microbial community stabilized into an altered, markedly depleted state, often described as an “alternative stable state” [11,32].

This phenomenon, often referred to as “microbiome scarring,” involves the persistent suppression or prolonged loss of highly specialized commensal microbes [11]. A key group affected by broad-spectrum antibiotics includes obligate anaerobes within the Firmicutes phylum, especially *Clostridium* clusters IV and XIVa. These clusters include important SCFA producers such as *Faecalibacterium prausnitzii*, *Roseburia*, *Coprococcus*, and *Anaerostipes* [33]. *F. prausnitzii*, a major butyrate-producing bacterium in a healthy colon, is very vulnerable to antibiotics and difficult to naturally recolonize afterward because of its strict, highly sensitive anaerobic growth requirements [33].

The depletion of butyrate-producing commensals serves as the primary molecular trigger for the structural breakdown of the intestinal epithelium in antibiotic-associated IBS models. Butyrate is more than just a byproduct of microbial carbohydrate fermentation; it is the main oxidative energy source for colonocytes and a powerful epigenetic regulator [23]. Mechanistically, butyrate acts as an endogenous inhibitor of histone deacetylases (HDACs) [23]. In the colonic epithelium, HDAC inhibition by microbial butyrate promotes an open, transcriptionally active chromatin state that is essential for the expression of key structural and barrier-strengthening genes [23].

The *SYNPO* gene is a key epigenetic regulatory target, encoding the actin-associated protein synaptopodin [23]. While it was first studied in renal podocytes where it helps regulate the actin cytoskeleton to prevent proteinuria by disrupting the Cdc42:IRSp53:Mena signaling complex [34], recent research has revealed its crucial role in maintaining intestinal epithelial barrier integrity [23]. In healthy conditions, microbiota-produced butyrate continuously stimulates high synaptopodin expression, which quickly localizes to the tight junctions of the intestinal epithelium and within F-actin stress fibers [23]. Synaptopodin functions to physically anchor and stabilize the perijunctional actomyosin ring, providing the mechanical tension and structural support needed to keep vital tight junction proteins like ZO-1 and Occludin securely attached to the lateral cell membranes [23,24,25].

When antibiotics damage the microbiome and reduce luminal butyrate production, the epigenetic signals that sustain *SYNPO* expression are quickly lost [23]. This causes a significant decrease in synaptopodin, destabilizing the intracellular F-actin network [23]. As a result, ZO-1 and Occludin lose their anchoring, leading to their endocytosis or abnormal redistribution away from the apical junctions [24,25]. Ex vivo studies of colonic biopsies from IBS patients consistently show this molecular defect: reduced mucosal levels and altered cellular localization of ZO-1 and Occludin, with degradation correlating with longer and more intense abdominal symptoms [24,25]. This structural failure results in a chronic, significant increase in paracellular permeability, often called a “leaky gut,” allowing luminal antigens, large molecules, and bacterial lipopolysaccharides (LPS) to pass freely into the sterile lamina propria [24,25].

### Factors Influencing Interindividual Ecosystem Resilience

Following antibiotic exposure, the degree and duration of microbiome recovery are highly variable between individuals. While microbial richness may considerably recover within several months in many individuals, specific taxonomic or functional alterations may remain in some subjects, driving a transition into significantly altered “alternative stable states” [35]. This variability in ecosystem resilience results from a complex interplay of host and environmental factors, beginning with baseline genetic diversity. High pre-existing microbial genetic diversity, including meta-gamma diversity from environmental reservoirs, provides a critical source of strains with differential sensitivities, facilitating structural re-equilibration after antibiotic-induced microbial depletion [35]. The composition and presence of keystone species prior to antibiotic treatment are essential for maintaining baseline stability. Keystone taxa, such as *Faecalibacterium prausnitzii* and *Bacteroides uniformis*, serve as fundamental metabolic drivers and primary colonizers, coordinating post-antibiotic ecological recovery of interdependent microbial groups. Their absence renders the ecosystem highly vulnerable to dramatic structural shifts [36].

Adult nutrition is also a significant contributing factor in these recovery results. High-fiber diets rich in microbiota-accessible carbohydrates provide alternative substrates for microbial fermentation, resulting in significant production of SCFAs, including butyrate, propionate, and acetate [37]. The availability of these SCFA substrates preserves the abundance of beneficial taxa and promotes Firmicutes resilience, therefore minimizing the severity of antibiotic collapse and accelerating recovery compared with fiber-deficient diets, which exacerbate microbial loss and substantially delay taxonomic restoration [35,37]. Ultimately, pre-existing psychological stress significantly reduces interindividual resilience by lowering baseline vagal tone, a key indicator of parasympathetic activity [38,39]. A strong baseline vagal tone physically buffers the gut by preserving mucosal barrier integrity, anti-inflammatory immunological tolerance, and microbial diversity [38,39]. Psychological stress reduces parasympathetic input, thereby increasing intestinal permeability and systemic inflammation [38,39]. As a result, stress-induced lowering of baseline vagal tone impairs critical neuroimmune-microbiome interaction, making the gut ecosystem more vulnerable to extensive and long-lasting dysbiosis when challenged with antibiotics [38,39]. Once this alternative, dysbiotic state is established, the loss of protective metabolites severely compromises the intestinal barrier. This structural failure sets the stage for the persistent immune activation and biochemical dysregulation detailed in Section 5.

## 5. Downstream Consequences: Immune Activation and Biochemical Disruption

The breakdown of the epithelial barrier due to microbiome scarring results in a persistent pathway for ongoing, low-level immune activation. Unlike the acute, neutrophil-dense, tissue-damaging inflammation seen in inflammatory bowel disease (IBD) or infectious enteritis, the inflammation in antibiotic-associated IBS is more subtle, highly localized, and predominantly mediated by the innate immune system’s peripheral sentinels: mucosal mast cells.

As paracellular permeability rises due to tight junction breakdown, bacterial antigens and LPS translocate and bind to Toll-like receptors (TLRs) on immune cells in the lamina propria. This persistent antigenic stimulation causes the recruitment, proliferation, and chronic activation of mucosal mast cells, which purposefully position themselves in extraordinarily close proximity to the sub-epithelial nerve plexuses [26,40]. When activated, these mast cells degranulate, releasing a neuroactive and pro-inflammatory mixture mainly composed of histamine, prostaglandin E2 (PGE2), and the serine protease tryptase [26,40,41].

Clinical evidence strongly supports this sequence. Biopsies from the rectosigmoid colon in patients with diarrhea-predominant IBS (IBS-D) reveal significant increases in mucosal mast cell density and a sharp rise in spontaneous tryptase and PGE2 release compared to healthy controls [40]. These mediators initiate a severe paracrine feedback loop that damages the gut barrier further and sensitizes the ENS. Notably, mast cell tryptase has a high affinity for Protease-Activated Receptor 2 (PAR2), a G protein-coupled receptor abundantly present on the basolateral membranes of colonocytes and the terminals of local enteric neurons [26].

When tryptase cleaves the extracellular N-terminus of PAR2, it exposes a new tethered ligand domain that folds back and binds to the receptor, triggering persistent intracellular signals [26]. In colonocytes, PAR2 activation links to β-arrestin-dependent pathways that activate the extracellular signal-regulated kinase 1/2 (ERK1/2) [26]. The phosphorylation of ERK1/2 leads to pathological changes in perijunctional F-actin, causing significant macromolecular paracellular flux [26]. Therefore, once initial barrier integrity is compromised, such as following antibiotic exposure, it sets off a self-perpetuating cycle of mast cell-driven barrier disruption that operates independently of the original trigger [26].

**Figure 1:**
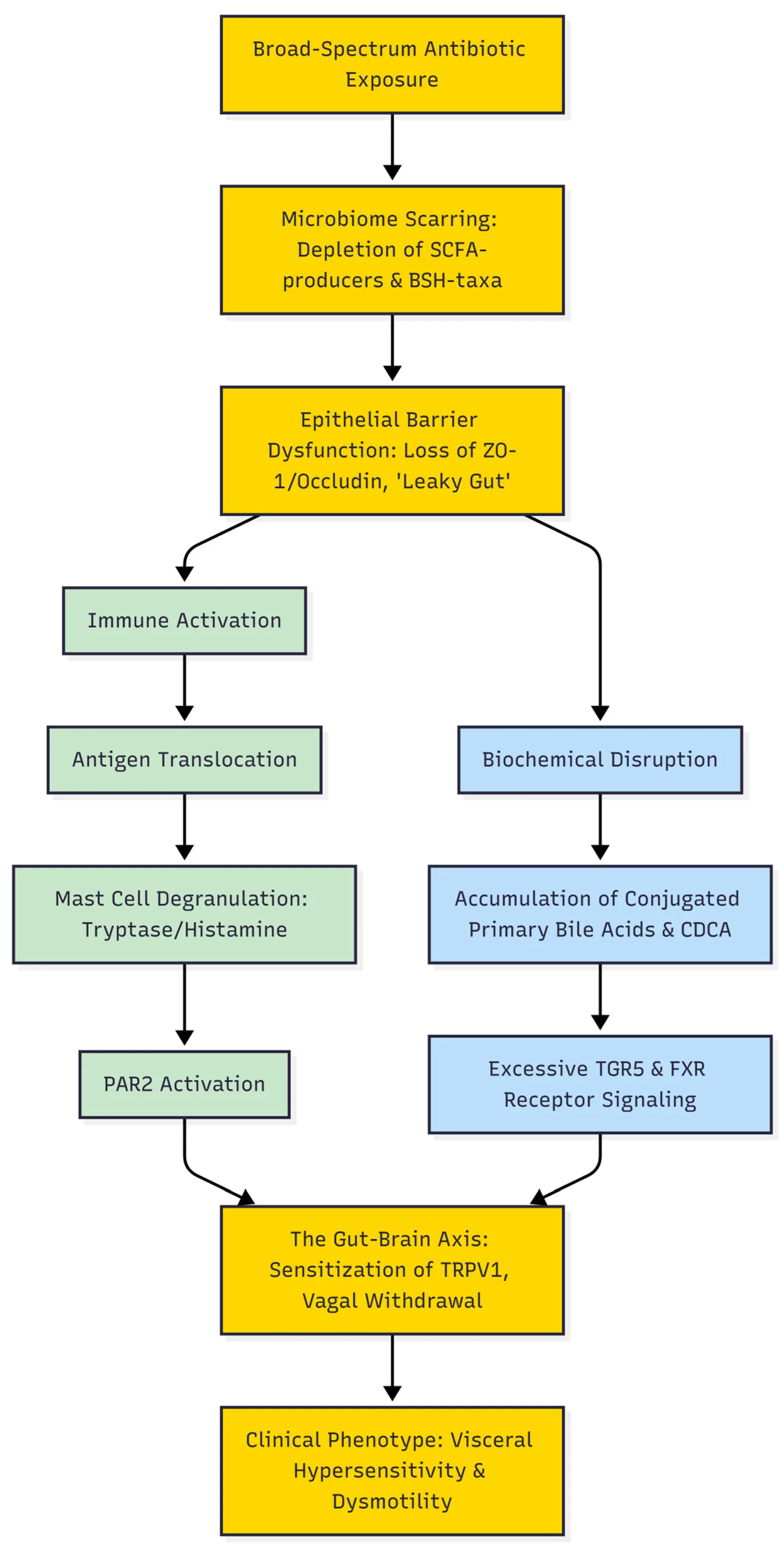
Mechanistic pathways linking broad-spectrum antibiotic exposure to the pathophysiology of irritable bowel syndrome (IBS). Antibiotic-induced microbiome scarring depletes short-chain fatty acid (SCFA)-producing and bile salt hydrolase (BSH)-expressing bacterial taxa, compromising epithelial tight junctions (ZO-1/Occludin) and inducing intestinal permeability. This barrier dysfunction triggers two parallel pathophysiological cascades: (1) an immune activation pathway characterized by antigen translocation, mast cell degranulation, and protease-activated receptor 2 (PAR2) activation; and (2) a biochemical disruption pathway driven by the luminal accumulation of conjugated primary bile acids and chenodeoxycholic acid (CDCA), leading to excessive TGR5 and FXR signaling. Both pathways converge on the gut–brain axis, promoting TRPV1 nociceptor sensitization and vagal withdrawal, which ultimately manifest as the clinical phenotype of visceral hypersensitivity and dysmotility.

With the transition of IBS from a purely “functional” label to a structurally grounded DGBI, the possibility of using histological evaluation for specific diagnosis has received much attention. Generally, standard hematoxylin and eosin (H&E) staining of colonic biopsies from IBS patients appears normal, which historically has contributed to the misclassification of IBS as a psychosomatic illness [42]. However, targeted quantitative histological evaluations have shown a consistent microscopic signature of low-grade mucosal inflammation. The most consistent finding in multiple studies is a significant increase in mucosal mast cell density and their increased proximity to enteric nerve fibers, particularly in the cecum and descending colon, often with increased intraepithelial lymphocytes (IELs) [42]. The quantification of mast cells with specific immunohistochemical stains (tryptase or CD117) is currently mostly a research tool and not a standardized clinical diagnostic criterion, because of histological overlap with other mild inflammatory states and healthy outliers [43]. However, the recognition of these unique micro-architectural and neuroimmune changes is an essential step toward the development of definitive diagnostic criteria in the future, the reduction in misdiagnosis, and the solidification of the structural pathophysiological basis of the disease. Therefore, the loss of epithelial integrity not only permits the translocation of immunogenic antigens but also facilitates the unregulated influx of luminal metabolites, driving a dual cascade of immune activation and biochemical disruption (Figure 1).

An imbalance in the gut flora will result in metabolic alterations. BAs are a vital component of the gut microbiota’s metabolism [44]. Abnormal BA metabolism is one of the typical manifestations in IBS-D patients, which is primarily characterized by an increase in fecal primary BA [44].

Gut bacteria possessing the enzyme Bile Salt Hydrolase (BSH) are responsible for deconjugating primary BAs by removing their amino acid conjugates. Subsequently, a distinct subset of bacteria with 7-alpha-dehydroxylase activity converts these newly unconjugated primary BAs into secondary forms, such as deoxycholic acid and lithocholic acid [45].

A healthy, homeostatic microbiome is particularly rich in BSH-producing bacteria, including species like *Bacteroides ovatus*, *Collinsella aerofaciens*, and various *Bifidobacterium* strains [44,45]. The antibiotic-associated eradication of these BSH-producing taxa creates a critical biochemical bottleneck. Without prior BSH-mediated deconjugation, 7-alpha-dehydroxylating bacteria are deprived of their necessary substrates. Consequently, this dysbiosis results in a severe depletion of secondary BAs and a substantial luminal accumulation of conjugated primary BAs (such as the glycine and taurine conjugates of CDCA) [44,45].

This altered luminal BA pool exerts profound, yet mechanistically distinct, physiological effects on the host via two separate receptor pathways. The Takeda G protein-coupled receptor 5 (TGR5) functions as a membrane-bound receptor that responds robustly to accumulating conjugated primary BAs; its hyperactivation on enterochromaffin cells increases the secretion of serotonin (5-HT), thereby driving the hypermotility and secretory pathophysiology characteristic of functional diarrhea (IBS-D) [44]. In contrast, the Farnesoid X Receptor (FXR) functions as a nuclear transcription factor that is stimulated by primary BAs such as CDCA. While FXR typically regulates systemic lipid and glucose homeostasis, its aberrant localized signaling in the mucosal mast cells mediates a distinct intracellular cascade that links primary BA accumulation directly to neurogenic visceral pain [44,46].

While the clinical pathways linking antibiotic use to constipation-predominant (IBS-C) and mixed (IBS-M) phenotypes remain complex, the unique microbiology of the gut provides a foundational clue. Methanogenic archaea, predominantly *Methanobrevibacter smithii*, possess a distinct phylogenetic structure that grants them innate resistance to a vast array of broad-spectrum antibiotics [47]. Because of this profound disparity in antimicrobial susceptibility, broad-spectrum therapies that readily eradicate vulnerable bacterial populations naturally spare these archaea [47]. Consequently, this innate survival advantage allows methanogens to withstand pharmaceutical treatments that severely alter the surrounding bacterial flora, creating a theoretical framework where they are perfectly positioned to survive and exploit the post-antibiotic ecological landscape [47]. Operating as an efficient hydrogenotroph, *M. smithii* sequesters the excess luminal hydrogen generated by surviving fermentative bacteria [48]. Rather than acting as an inert metabolic byproduct, accumulated methane functions as a biologically active gasotransmitter. In preclinical models, methane acts as a local neuromuscular modifier within the ENS; it directly augments small intestinal contractile activity, enhancing non-propulsive segmental smooth muscle contractions that significantly slow overall intestinal transit [49]. Translating these motility changes into clinical practice, modern consensus guidelines now formally recognize the expansion of these methane-producing archaea as a distinct clinical entity. Officially categorized as Intestinal Methanogen Overgrowth (IMO), this condition is now firmly established as a measurable driver of IBS-C and IBS-M phenotypes, differentiating it from traditional bacterial overgrowth and necessitating targeted diagnostic breath testing to guide clinical management [50].

### Distinguishing the Dysbiotic Phenotype: IBS Versus IBD

The “leaky gut” phenomenon, microbial dysbiosis, mucosal injury, and altered cytokine milieu are well-established hallmarks of IBD, including Crohn’s disease and ulcerative colitis. Therefore, it is critical to distinguish the pathophysiological trajectory of IBD from that of IBS [51]. Both conditions can be precipitated or worsened by antibiotic-associated ecological disruption, but they differ fundamentally in the magnitude, localization, and cellular nature of the inflammatory response. The disruption of the epithelial barrier in IBD leads to severe, destructive infiltration of both the innate and adaptive immune systems, dominated by neutrophils, macrophages, and T-lymphocytes. This leads to macroscopic architectural distortion, crypt abscesses, deep mucosal ulceration, and a severe systemic cytokine storm (e.g., elevated TNF-α and IL-6) [51]. In contrast, the antibiotic-associated mucosal inflammation in IBS is strictly microscopic and low-grade. The gross mucosal architecture is entirely intact with no macroscopic ulceration or crypt branching. In addition, the inflammatory infiltrate in IBS is mostly neuroimmune and innate in nature, as evidenced by the almost exclusive localized proliferation and degranulation of mast cells and eosinophils in close proximity to the enteric nerve endings [52]. IBD is characterized by continuous, progressive tissue destruction, while IBS is characterized by localized neurogenic dysfunction and visceral hypersensitivity due to subtle, but persistent, mucosal signaling. Crucially, these localized immune and metabolic disruptions do not remain confined to the mucosa. Section 6 delineates how these inflammatory signals are transmitted via the gut–brain axis, ultimately manifesting as visceral hypersensitivity.

## 6. The Gut–Brain Axis: Translating Localized Disruption to Clinical Symptoms

The most severe and disabling manifestation of IBS is visceral hypersensitivity, a pathological condition where normal physiological processes, such as gas transit or baseline peristalsis, are perceived by the CNS as intense, localized abdominal pain. The proposed conversion of localized antibiotic-induced dysbiosis into severe neurological pain is regulated by the highly interconnected circuits of the gut–brain axis, mediated through the interaction among BAs, neurotrophic factors, and sensory ion channels.

The connection between BA accumulation and neurogenic pain is primarily mediated by the FXR. Recent research has demonstrated that when primary BAs, such as CDCA, accumulate and excessively stimulate FXR on colonic mucosal mast cells, this receptor induces a strong transcriptional response and significant localized secretion of Nerve Growth Factor (NGF) [16]. This FXR-dependent expression of NGF is contingent upon the activation of upstream intracellular signaling pathways, including MKK3/6, p38 MAPK, and NF-κB, within the mast cell cytoplasm [16].

Upon entry into the lamina propria, NGF functions as a highly potent neuromodulator. It exhibits high-affinity binding to its specific receptor, Tropomyosin receptor kinase A (TrkA), which is extensively expressed on the peripheral terminals of unmyelinated nociceptive spinal afferents (C-fibers) innervating the gut wall [53]. The interaction between NGF and TrkA triggers a substantial intracellular signaling cascade within the sensory neuron that ultimately leads to marked phosphorylation and heightened sensitization of Transient Receptor Potential Vanilloid 1 (TRPV1) ion channels located on the neuronal membrane [16,53].

TRPV1 serves as the primary molecular transducer for noxious stimuli and inflammatory pain within the viscera [16]. In an unaltered, healthy state, colonic TRPV1 channels necessitate substantial mechanical stretch, significant thermal variations, or highly aggressive chemical stressors to become activated and depolarize the neuron. However, following phosphorylation via the NGF/TrkA pathway, the activation threshold of TRPV1 is markedly and persistently reduced. Consequently, the channel is capable of initiating action potentials in response to normal, non-noxious colonic distension, driving the clinical presentation of visceral hypersensitivity [16,53]. These hyperactive nociceptive signals are transmitted rapidly via the dorsal root ganglia to the central autonomic network in the brain, culminating in the persistent abdominal pain characteristic of IBS.

Meanwhile, the bidirectional communication within the gut–brain axis fails to mitigate hypersensitivity due to impairment of the vagus nerve. As the main pathway of the parasympathetic nervous system, the vagus nerve possesses significant anti-inflammatory and barrier-preserving functions across the gastrointestinal tract through the Cholinergic Anti-inflammatory Pathway (CAP) [54]. Under physiological stress, efferent vagal fibers release acetylcholine, which binds to α7 nicotinic acetylcholine receptors (α7nAChR) on tissue-resident macrophages, actively reducing pro-inflammatory cytokine secretion and maintaining the integrity of tight junctions [54,55].

Nevertheless, persistent low-grade mucosal inflammation, ongoing afferent pain signaling to the brainstem, and associated psychological stress resulting from chronic IBS effectively suppress vagal tone [54]. This pronounced vagal withdrawal precipitates systemic failure of the CAP, leading to unchecked sympathetic autonomic overactivity [54]. The significant reduction in vagal regulation further worsens paracellular permeability, notably causing a substantial decrease in Occludin expression and morphological alterations in enteric glial cells, thereby entrenching a neurologically mediated vicious cycle wherein the gut’s inability to heal is perceived as continuous stress by the brain, while the brain’s perception of persistent stress is driven by gut barrier failure [54]. A comprehensive summary of these interconnected mechanistic pathways, detailing the progression from initial microbiome scarring to downstream clinical phenotypes, is provided in Table 2. Understanding this complete cascade underscores the profound impact of antibiotic overuse. Consequently, Section 7 outlines the critical clinical implications of these findings, focusing on antimicrobial stewardship and emerging microbiome-sparing interventions.

## 7. Clinical Implications and Future Directions

Recognition of systemic antibiotics as a potential, dose-dependent, and modifiable predisposing factor for IBS necessitates a fundamental shift in practices across primary care and gastroenterology. Understanding that standard prescriptions for mild infections pose a significant risk for predisposing individuals to chronic disorders related to gut–brain interactions highlights the urgent need for rigorous antimicrobial stewardship. Future gastroenterological approaches should emphasize the implementation of prophylactic microbiome-preserving strategies during antibiotic treatment and the development of targeted therapeutics to mitigate iatrogenic harm.

### 7.1. Prophylactic Microbiome-Sparing Strategies

**Figure 2:**
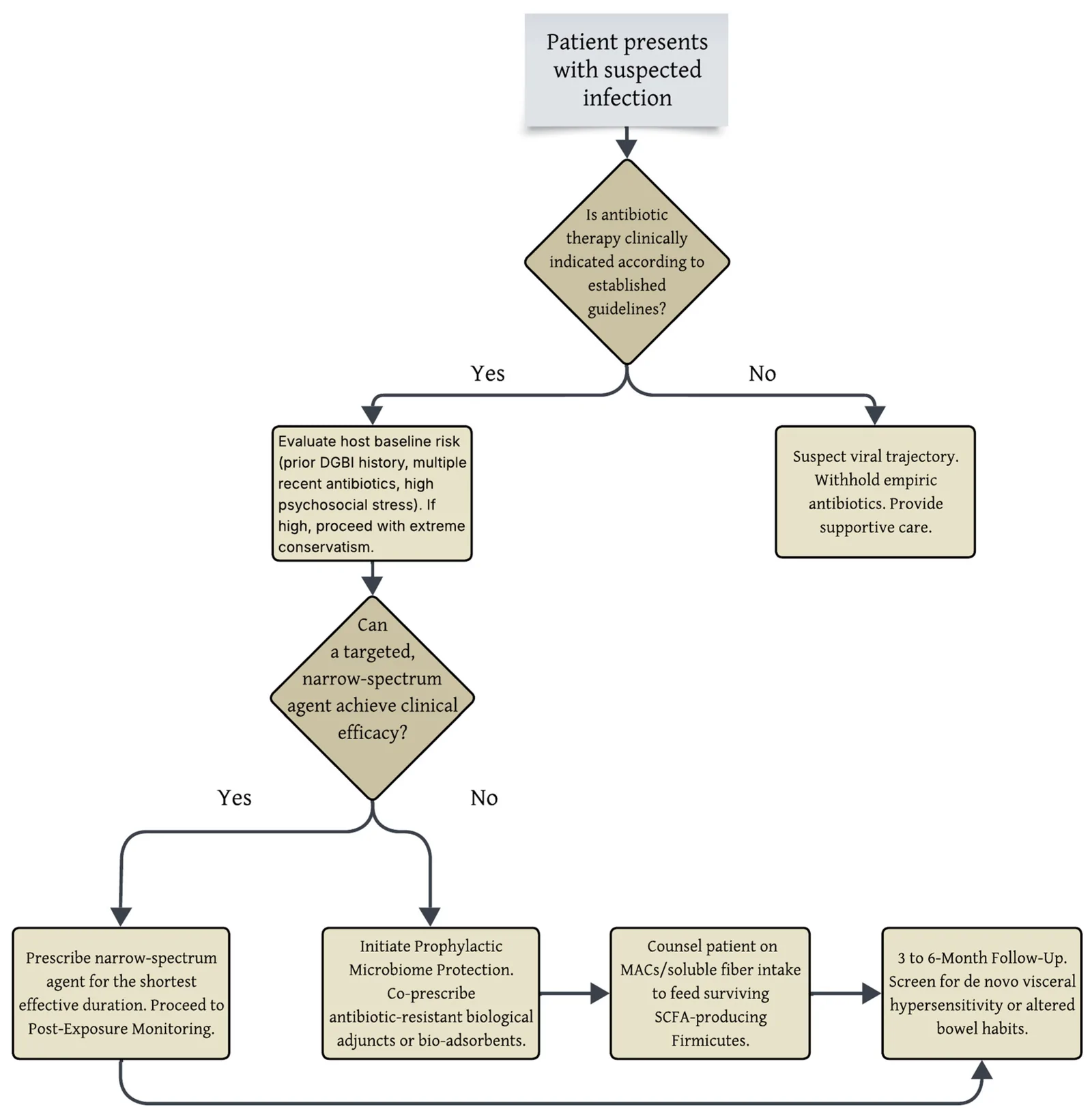
DGBI-Risk Mitigation Clinical Decision Tree. This algorithmic pathway provides a stepwise framework for host risk stratification, precision antimicrobial selection, and prophylactic microbiome-sparing interventions to minimize iatrogenic dysbiosis. Clinical Disclaimer: This algorithm is investigational and intended solely as a supplementary framework for stable, non-critical patients; it does not replace established infectious disease or antimicrobial stewardship guidelines. The primary clinical decision must always be whether empiric or targeted antibiotic therapy is clinically indicated according to current guidelines, rather than whether definitive microbiological confirmation is already available. It is contraindicated in cases of sepsis, immunodeficiency, neutropenia, meningitis, severe pneumonia, or any emergency setting. Abbreviations: DGBI, Disorders of Gut–Brain Interaction; MACs, Microbiota-Accessible Carbohydrates; SCFA, Short-Chain Fatty Acids.

To diminish the ecological impact of broad-spectrum antibiotics, clinical interventions must either sequester the antimicrobial agent from the colonic ecosystem or employ adjunctive biological agents that preserve microbiome metabolic function during treatment. To support these stewardship objectives, a clinical decision algorithm for DGBI risk mitigation is proposed for use prior to initiating empiric systemic antibiotics (Figure 2). It is important to emphasize that this framework is intended solely as a supplementary risk-mitigation tool for stable patients in non-emergent settings and does not replace established infectious disease or antimicrobial stewardship guidelines. The primary clinical decision must always be whether empiric or targeted antibiotic therapy is clinically indicated according to current guidelines, rather than whether definitive microbiological confirmation is already available. This algorithm should not be used in patients presenting with sepsis, immunodeficiency, neutropenia, meningitis, severe pneumonia, or any acute clinical emergency. Moreover, all proposed microbiome-protective interventions in this algorithm are to be considered investigational.

One of the most promising advancements in this domain is DAV132, a novel colon-targeted bio-adsorbent derived from microencapsulated activated charcoal [57,58]. Developed with an advanced polymer coating designed to bypass the stomach and small intestine, DAV132 releases highly potent activated charcoal specifically in the late ileum and colon [57]. In rigorous Phase I and Phase II clinical trials involving both healthy volunteers and hospitalized patients undergoing intensive fluoroquinolone and beta-lactam therapy, the co-administration of DAV132 resulted in a reduction in free fecal antibiotic concentrations by over 98% [57,58]. Importantly, this sequestration occurred without affecting the therapeutic plasma levels necessary for systemic infection clearance [58]. By sequestering antibiotic residues in the lower gastrointestinal tract, DAV132 maintained the taxonomic richness and beta-diversity of the gut microbiome, thereby preventing antibiotic-induced dysbiosis and subsequent barrier failure [57,58]. However, while DAV132 successfully preserves microbiome diversity in clinical settings, prospective trials are still needed to confirm whether this ecological preservation directly translates to the prevention of incident IBS.

The concurrent administration of highly selective, non-bacterial probiotics offers a robust biological mechanism for ecosystem buffering. *Saccharomyces boulardii* CNCM I-745, a probiotic medicinal yeast, is considered an exemplary candidate for prophylactic use [59,60]. As a eukaryotic organism, *S. boulardii* exhibits innate resistance to all classes of antibacterial agents, enabling it to maintain functional activity and metabolic vitality during ongoing antibiotic treatment [60]. Extensive clinical trials involving humans and studies with murine models demonstrate that *S. boulardii* promotes the rapid restoration of intestinal microbiota following significant antibiotic-associated disruption [59]. Importantly, it facilitates the proliferation of SCFA-producing bacteria, such as those within the *Lachnospiraceae* and *Ruminococcaceae* families, while simultaneously inhibiting the overgrowth of pathogenic pioneer species [59]. By stabilizing the production of immunoregulatory metabolites like propionate and butyrate, *S. boulardii* plays a crucial role in maintaining epigenetic regulation essential for barrier homeostasis, thereby reducing the risk of progression to chronic disease states [59,60]. Nevertheless, although this probiotic effectively mitigates acute taxonomic alterations, direct clinical evidence demonstrating its ability to prevent the long-term development of antibiotic-associated IBS is currently lacking.

### 7.2. Potential and Investigational Therapeutic Approaches

Because it is currently difficult to differentiate antibiotic-induced dysbiosis from other overlapping functional and psychosocial factors in a clinical setting, management should integrate standard, symptom-directed IBS therapies alongside investigational strategies that aim to directly target the proposed structural and biochemical abnormalities.

To counteract the pronounced depletion of endogenous butyrate and the consequent deterioration of synaptopodin-dependent barrier integrity, the administration of colon-specific, microencapsulated sodium butyrate has demonstrated significant clinical effectiveness. Given that unprocessed oral butyrate is rapidly absorbed in the proximal gastrointestinal tract and has a notably unpleasant odor, advanced formulations using triglyceride-matrix and nanogel encapsulation techniques have been developed to enable targeted, sustained delivery directly to the colonic mucosa [61,62]. In extensive, prospective, multicenter clinical trials involving thousands of individuals diagnosed with IBS, the administration of targeted microencapsulated sodium butyrate markedly alleviated symptoms including abdominal pain, flatulence, and abnormal bowel habits over a 12-week period [61,62]. By replenishing the luminal SCFA pool, these formulations emulate the epigenetic signaling typically mediated by commensal bacteria such as *F. prausnitzii*, thereby maintaining HDAC inhibition, promoting *SYNPO* gene expression, and restoring the integrity of paracellular tight junctions from within the epithelium [23]. It should be noted, however, that the current clinical evidence for microencapsulated butyrate is derived from general IBS populations; its specific efficacy within a confirmed antibiotic-associated IBS cohort requires further targeted validation.

Addressing the biochemical dysregulation of BAs is equally essential for achieving long-term symptom resolution. BA sequestrants, such as the gut-restricted resin colesevelam, have demonstrated efficacy in the management of IBS-D and BA diarrhea [63]. By physically binding excess primary BAs within the lumen, colesevelam significantly elevates overall fecal BA excretion [63]. This sequestration results in slowed colonic transit, increased stool consistency, and active reduction in BA-induced increases in mucosal permeability [63]. The direct pharmacological modulation of FXR has also been proposed; however, its application in IBS is highly complex. For example, while gut-restricted FXR agonists like fexaramine have shown efficacy in models of obesity and metabolic syndrome, their physiological effects differ significantly depending on the specific ligand, the targeted tissue, the luminal BA profile, and the underlying disease context [64]. In the setting of antibiotic-associated IBS, exogenous and non-selective FXR agonism could theoretically exacerbate the visceral pain pathways previously described rather than resolving them. Therefore, while the BA sequestrant colesevelam is an established intervention for BA diarrhea and general IBS-D, its therapeutic utility has not yet been prospectively evaluated specifically in patients with antibiotic-associated dysbiosis.

Pharmacological mitigation of the hypersensitive gut–brain axis necessitates intervention at the mucosal interface to disrupt the mast cell-to-neuron signaling cascade. The use of potent mast cell stabilizers such as ketotifen has been shown to markedly elevate the sensory threshold for visceral discomfort in patients with IBS who display visceral hypersensitivity [41]. By inhibiting the degranulation of tryptase and histamine, ketotifen effectively prevents PAR2 activation in nociceptors and inhibits subsequent phosphorylation of TRPV1 channels, thereby reducing the peripheral initiation of pain signals [26,40]. Although ketotifen has demonstrated efficacy in mitigating visceral hypersensitivity in general IBS populations, targeted trials evaluating its utility specifically in an antibiotic-associated subgroup are presently lacking.

The restoration of the gut’s impaired autonomic regulation through bioelectronic medicine is emerging as a promising, non-pharmacological therapeutic approach. Transcutaneous Vagus Nerve Stimulation (tVNS), applied non-invasively via the auricular concha, directly activates the CAP [55]. In clinical settings, brief sessions of tVNS have been demonstrated to suppress stress-induced intestinal permeability, protecting barrier integrity as evidenced by preserved physiological biomarkers [55]. By counteracting sympathetic overactivity induced by chronic visceral stress, tVNS links macro-level neurological dysfunction to micro-level mucosal repair, thereby offering a comprehensive approach to healing the gut–brain axis [54,55]. At present, tVNS represents a theoretical investigational approach based on broader neuromodulatory principles; there is currently no direct clinical evidence demonstrating its efficacy for treating antibiotic-associated IBS.

### 7.3. Critical Appraisal, Confounding Factors, and Controversies

While the evidence linking antibiotic exposure to IBS is compelling, a balanced critical appraisal reveals significant controversies. A major epidemiological challenge is confounding by indication; it is often difficult to definitively separate the pathophysiological impact of the antimicrobial agent from the underlying infection that prompted its prescription [13,14]. Although some studies attempt to mitigate this by examining non-enteric infections, residual confounding from the host’s response to the infection itself cannot be entirely eliminated [14]. Furthermore, current epidemiological literature frequently lacks robust adjustments for critical lifestyle confounders, such as baseline dietary fiber intake and underlying psychosocial stress, both of which independently dictate microbiome resilience and IBS risk [35,38].

Moreover, the concept of permanent “microbiome scarring” is not universally supported. Conflicting longitudinal evidence demonstrates that in a substantial subset of healthy individuals, the microbiome exhibits remarkable elasticity, recovering its near-baseline taxonomic composition and metabolic capacity within 1.5 to 6 months of antibiotic cessation without precipitating functional bowel symptoms [65].

Therefore, there are limitations to the current evidence that must be recognized. A primary limitation is that “antibiotic-associated IBS” currently lacks universally accepted diagnostic criteria, validated biomarkers, and a prospectively confirmed clinical phenotype. The pathways described in this paper serve as a hypothesis-generating pathophysiologic framework rather than a definitive clinical diagnosis. Moreover, as a narrative review, this manuscript relies on curated literature rather than a systematic quantitative synthesis. Finally, important translational gaps remain in the foundational literature. Mechanistic insights are often based on in vitro and murine models, necessitating careful interpretation given species-specific host-microbiome dynamics. Furthermore, generalizing data from an acute ecological collapse (such as *C. difficile* infection) to the chronic, low-grade inflammatory state of IBS carries inherent limitations, as the taxonomic shifts and mucosal alterations in IBS are far more subtle.

### 7.4. Knowledge Gaps and Future Research Priorities

To transition microbiome-sparing strategies from theoretical frameworks to evidence-based clinical guidelines, future research must address several critical knowledge gaps. Currently, most clinical data on interventions like DAV132 and *S. boulardii* focus on preventing general dysbiosis or acute antibiotic-associated diarrhea [57,59]. Their efficacy in the long-term prevention of incident IBS remains extrapolated. Therefore, a primary priority is the initiation of large-scale, prospective, interventional clinical trials with extended follow-up (e.g., 12 to 24 months) to evaluate whether co-administering these agents definitively reduces the epidemiological incidence of DGBIs.

Additionally, future study designs must incorporate longitudinal multi-omics (metagenomic and metabolomic) profiling to identify predictive biomarkers of vulnerability [66]. Identifying specific baseline microbial signatures that predispose an individual to irreversible ecological disruption would allow clinicians to stratify patient risk and apply precision-medicine approaches to antimicrobial stewardship. Ultimately, closing these translational gaps is essential to validate antibiotic-associated IBS as a distinct clinical entity and to establish prophylactic protocols that protect the gut–brain axis.

## 8. Conclusions

The growing understanding of DGBIs needs a fundamental paradigm shift in the clinical assessment of systemic antibiotic treatment. Broad-spectrum antibiotics are not harmless, temporary therapies; rather, they are strong ecological disruptors that cause substantial “microbiome scarring”, acting as a potential, dose-dependent predisposing factor for incident IBS. This iatrogenic dysbiosis initiates a predictable pathogenic cascade: the removal of SCFA-producing taxa reduces *SYNPO*-mediated epithelial barrier integrity, whereas altered BA metabolism and persistent mucosal neuroimmune activation chronically sensitize the gut–brain axis. Recognizing this significant collateral damage reinforces an urgent clinical imperative for stricter antimicrobial stewardship and highlights promising investigational approaches, such as targeted bio-adsorbents and microbiome-sparing probiotics, that require prospective clinical validation before routine implementation to help mitigate the growing global burden of antibiotic-associated functional bowel disorders.

## Figures and Tables

**Table 1:** Core epidemiological evidence on antibiotic-associated IBS and associated risk metrics.

Author	Study Design & Diagnostic Criteria	Key Variables & Study Limitations	Core Findings & Risk Metrics
Krogsgaard et al. [12]	Prospective web-based cohort survey (*n* = 2781). Criteria: Rome III criteria. Exclusions: Structural GI diagnosis.	Variables: Baseline asymptomatic controls tracked longitudinally over 3 years. Limitations: Self-reported antibiotic use (recall bias); lacked data on specific antibiotic class and indication.	22.4% reported antibiotic use. Antibiotic exposure predicted incident IBS with a RR of 1.9 (95% CI: 1.1 to 3.1). Adjusted OR for sex was 1.8 (95% CI: 1.0 to 3.2).
Staller et al. [13]	Nationwide case–control (29,111 IBS cases vs. 135,172 matched controls) Criteria: First-ever IBS diagnosis via ICD codes, requiring a lifetime colonoscopy biopsy. Exclusions: Prior diagnosis of IBD, Celiac disease, or colorectal cancer.	Variables: Cumulative antibiotic dispensations up to 1 year prior to IBS diagnosis (exclusionary buffer). Limitations: Dispensation data does not guarantee actual patient consumption; lacked data on clinical indications for antibiotics; potential selection bias due to the colonoscopy requirement.	74.9% of IBS patients had prior antibiotic exposure vs. 57.8% of controls. Overall adjusted OR for IBS was 2.21 (95% CI: 2.14 to 2.28). Demonstrated strict dose-dependency: 1–2 prescriptions OR 1.67 (95% CI: 1.61 to 1.73); ≥3 prescriptions OR 3.36 (95% CI: 3.24 to 3.49) (*p* < 0.001).
Paula et al. [14]	Nested case–control study derived from prospective longitudinal surveys (316 new-onset FGID cases vs. 250 controls). Criteria: Modified Rome II criteria using the Mayo Bowel Disease Questionnaire. Exclusions: Individuals reporting any FGID symptoms at the baseline survey were excluded.	Variables: Blinded chart reviews assessed history of enteric vs. non-enteric infections and antibiotic usage prior to symptom onset. Limitations: Unable to determine the precise temporal relationship between antibiotic exposure and FGID onset within the survey intervals; potential bias from healthcare-seeking behavior; lacked diagnostic workup to definitively exclude structural dyspepsia.	83% of new FGID cases who had a non-GI infection were treated with antibiotics. Antibiotic treatment for a non-gastrointestinal infection was an independent predictor for developing a subsequent FGID (Adjusted OR 1.90; 95% CI: 1.21 to 2.98; *p* = 0.005). Rates of actual GI infections were similar between both groups, isolating the non-enteric antibiotic variable.
Colecchia et al. [18]	Systematic review and meta-analysis (31 studies, *n* = 422,350; 244,632 antibiotic users vs. 177,718 nonusers). Criteria: Studies reporting new diagnoses of IBS in patients with documented antibiotic exposure versus controls without antibiotic exposure.	Variables: Pooled incidence rates and IRRs; evaluated the impact of geographical area, diagnostic criteria, and study quality via metaregression analysis. Limitations: High significant heterogeneity among the included studies (*I*^2^ > 90%), which reduces the statistical power of the pooled results.	Overall pooled incidence of IBS was 26% in antibiotic users compared to 20% in nonusers. Overall IRR was 1.30 (95% CI: 1.07 to 1.58, *p* = 0.008). Risk was notably higher when antibiotics were used specifically for gastrointestinal infections (IRR 1.71; 95% CI: 1.16 to 2.51, *p* = 0.007).

Abbreviations: CI, Confidence Interval; FGID, Functional Gastrointestinal Disorder; GI, Gastrointestinal; IBD, Inflammatory Bowel Disease; IBS, Irritable Bowel Syndrome; ICD, International Classification of Diseases; IRR, Incidence Rate Ratio; OR, Odds Ratio; RR, Relative Risk.

**Table 2:** Summary of the molecular, cellular, and neuroimmune mechanisms driving antibiotic-associated IBS.

Upstream Trigger (Iatrogenic Event)	Proposed Microbial Mechanism	Downstream Neurological/Clinical Effect	Population/Model	Level of Evidence	Relevance to Antibiotic-Associated IBS	Reference
Depletion of *F. prausnitzii* & Firmicutes	Reduction in luminal Butyrate → Loss of HDAC inhibition → *SYNPO* gene downregulation → ZO-1/Occludin destabilization.	Loss of barrier integrity; increased paracellular permeability; facilitating antigen translocation.	In vitro (T84 human colonic cells, murine enteroids) & In vivo (Antibiotic-depleted mice, DSS colitis models).	Preclinical	Hypothetical (Extrapolated from IBD/colitis and general barrier dysfunction models).	[23]
Translocation of LPS & Antigens	Chronic activation of lamina propria mucosal mast cells → Degranulation of Tryptase, Histamine, and PGE2.	Tryptase cleaves PAR2 on nerves and colonocytes, inducing epithelial disruption and directly lowering firing thresholds of enteric sensory nerves.	Ex vivo (Human IBS mucosal biopsies).	Ex vivo Clinical	Supportive (The PAR2 pain pathway is definitively established in human IBS models).	[27]
Eradication of *Bacteroides ovatus* & BSH taxa	Loss of *Bacteroides ovatus* and BSH-producing taxa reduces bile acid deconjugation, leading to luminal accumulation of conjugated primary bile acids.	Conjugated primary bile acids hyper-activate TGR5 receptors on enterochromaffin cells, inducing colonic barrier dysfunction (decreased Claudin-1, E-cadherin), serotonin (5-HT) release, and accelerated intestinal transit.	Human (IBS-D cohorts); In vivo (Microbiota-humanized rats, CDCA-gavaged rats, and antibiotic-treated murine models).	Clinical Observational & Preclinical	Supportive (Antibiotics definitively deplete BSH taxa; the resulting CDCA/TGR5 axis drives the IBS-D phenotype).	[44]
BA (CDCA) Overload	Accumulation of primary BAs such as CDCA activates FXR on mucosal mast cells, triggering p38 MAPK/NF-κB signaling and the release of NGF.	NGF binds TrkA on DRG nociceptors, upregulating TRPV1 to cause visceral hypersensitivity.	In vitro/In vivo (BA-infused rodents and human/rat mast cell lines).	Preclinical	Supportive (The FXR-NGF pain cascade is established in preclinical BA-infusion models).	[16]
Chronic Intestinal Stress & Inflammation	Stress induces CRH, altering the barrier. Mucosal mast cells and resident macrophages release pro-inflammatory cytokines, NO, and prostaglandins.	Breakdown of tight junctions creates a “leaky gut”. Inflammatory mediators sensitize spinal afferent nerves and impair smooth muscle contractility, driving dysmotility.	Human (IBS/IBD cohorts, healthy volunteers).	Clinical Observational	Supportive (Barrier dysfunction via CRH/vagal pathways is established in functional bowel disorders).	[56]
Depletion of competitive bacterial clades	Disruption of normal flora is hypothesized to enable Intestinal Methanogen Overgrowth (IMO), predominantly *Methanobrevibacter smithii*. These archaea consume hydrogen to produce luminal methane gas.	Methane gas directly alters enteric neuromuscular function by augmenting segmental, non-propagating contractions, delaying peristaltic conduction and slowing transit.	Human (Methane-producing IBS-C cohorts); In vivo (Canine transit models, guinea pig ileum).	Preclinical & Clinical Observational	Hypothetical (Provides a potential link between microbial dysbiosis, overgrowth of gas-producing archaea, and altered intestinal motility, though direct clinical progression from antibiotic exposure requires further validation).	[49,50]

Abbreviations: BA, Bile Acid; BSH, Bile Salt Hydrolase; CDCA, Chenodeoxycholic Acid; CRH, Corticotropin-Releasing Hormone; DRG, Dorsal Root Ganglion; DSS, Dextran Sulfate Sodium; FXR, Farnesoid X Receptor; HDAC, Histone Deacetylase; IBD, Inflammatory Bowel Disease; IBS, Irritable Bowel Syndrome; IBS-C, Constipation-Predominant Irritable Bowel Syndrome; IBS-D, Diarrhea-Predominant Irritable Bowel Syndrome; IMO, Intestinal Methanogen Overgrowth; LPS, Lipopolysaccharide; MAPK, Mitogen-Activated Protein Kinase; NF-κB, Nuclear Factor kappa B; NGF, Nerve Growth Factor; NO, Nitric Oxide; PAR2, Protease-Activated Receptor 2; PGE2, Prostaglandin E2; SYNPO, Synaptopodin; TGR5, Takeda G protein-coupled receptor 5; TrkA, Tropomyosin receptor kinase A; TRPV1, Transient Receptor Potential Vanilloid 1; ZO-1, Zonula Occludens-1.

## Data Availability

No new data were created or analyzed in this study. Data sharing is not applicable.
